# Understanding trends in osteoporosis drug prescribing: implications for reducing futile biomedical research

**DOI:** 10.3389/fmed.2024.1454150

**Published:** 2025-01-13

**Authors:** Jonathan R. Guillemot, John W. Abraham, Anthea Tinker

**Affiliations:** ^1^Escuela de Medicina, Instituto de Medicina Social and Desafíos Globales, Colegio de Ciencias de la Salud, Universidad San Francisco de Quito, Quito, Ecuador; ^2^Department of Global Health and Social Medicine, Institute of Gerontology, Faculty of Social Science and Public Policy, King’s College London, London, United Kingdom; ^3^Centre for Research in Health and Medicine, Sussex Medical School, University of Sussex, Brighton, United Kingdom

**Keywords:** osteoporosis, geriatrics, pharmaceuticalization, medicalization, prescription, older adults

## Abstract

**Introduction:**

Osteoporosis is a significant geriatric condition, considering its impact on fracture-related morbidity and mortality, particularly among older women. The interplay of clinical evidence, diagnostic tools availability, and broader societal attitudes toward aging and treatment efficacy affect medical attitude and prescribing behaviors. Using the example of osteoporosis in France and England, the study aims to unravel the intricacies of medical decision-making in geriatric care, offering insights into the evolving landscape of healthcare policy and practice, which in turn can help reduce futile biomedical research.

**Methods:**

We employed documentary analysis and semi-structured interviews. Documentary analysis involved examining public policy documents related to osteoporosis management in England and France to identify trends in regulatory policies influencing prescribing practices. Semi-structured interviews with physicians explored prescriber decision-making processes, treatment initiation, and compliance management, providing insights into clinical practice complexities.

**Results:**

The policy analysis uncovered 157 documents between 2015 and 2016, updated in 2018, revealing distinct policy clusters and outliers shaping osteoporosis management in England and France. Therapeutic indications generally mirrored marketing authorizations. Reimbursable therapeutic indications in France showed fluctuating availability, reflecting changes in policy priorities and patient demographics. Clinical guidelines evolved to encompass diverse osteoporosis types and treatment options, guided by evidence-based recommendations and healthcare system considerations. Trust dynamics between physicians, pharmaceutical companies, and health authorities influenced prescribing trends, with variations in reliance on standardized protocols and collaborative decision-making observed between England and France.

**Discussion:**

Understanding trends in osteoporosis drug prescribing is crucial for optimizing healthcare policy and practice. Our study highlights the complex factors influencing prescribing patterns in England and France, emphasizing the role of trust in shaping physician behaviors. By addressing barriers to treatment uptake and enhancing patient outcomes, targeted interventions can be developed to reduce futile biomedical research and improve healthcare resource allocation.

## 1 Introduction

Osteoporosis, emblematic of geriatric conditions, epitomizes the intricate intersection of attitudes toward care and treatment trends in aging populations. Predominantly affecting older women, osteoporosis manifests as bone fragility, predisposing individuals to fractures, notably from low-impact falls (1). While largely asymptomatic, osteoporosis poses a significant risk for fracture-related morbidity and mortality. Despite its prevalence in older age groups, osteoporosis transcends age boundaries, impacting individuals as young as 50 years. Hormonal changes, particularly post-menopause, amplify the susceptibility to osteoporosis in women, underscoring the multifactorial nature of its etiology (2).

Medical attitudes toward osteoporosis have evolved over time, from its historical perception as a natural consequence of aging to its recognition as a serious health concern necessitating intervention (2). The emergence of diagnostic criteria, notably bone mineral density measurements via DEXA scans (dual-energy X-ray absorptiometry), revolutionized osteoporosis diagnosis and management, albeit amid controversies surrounding diagnostic thresholds. This led to the emergence of the concept of osteopenia, defined as preliminary osteoporosis, but which is often addressed as a medical condition, as such. This evolution in diagnostic techniques was accompanied by a parallel expansion in therapeutic options, facilitated by the pharmaceutical industry’s heavy investment in diagnostic technologies and drug development (3). However, the narrative of osteoporosis extends beyond medical discourse, intersecting with broader societal debates on disease mongering, pharmaceutical influence as well as attitudes toward physiological aging. Criticisms of medicalization and pharmaceuticalization have fueled skepticism toward pharmaceutical interventions, questioning the balance between genuine healthcare needs and commercial interests (4–6).

Amidst these complexities, age bias emerges as a significant determinant of osteoporosis management (7). The interplay between medicalization, pharmaceutical influence, and societal perceptions underscores the need for a nuanced understanding of osteoporosis trends and their implications for aging research and healthcare policy. In the realm of biomedical research, the pursuit of scientific advancement is often hindered by inefficiencies and wasteful practices that undermine the potential impact of research endeavors. Authors such as Paul Glasziou and Iain Chalmers have extensively documented the pervasive issue of waste in biomedical research, estimating that a staggering 85% of global research investment is squandered annually (8). Such waste spans every stage of the research process, from the formulation of research questions to the dissemination of results and raises profound questions regarding efficiency of health investments. One area particularly susceptible to wasteful practices is the prescribing of pharmaceutical treatments, where suboptimal decision-making and overmedicalization contribute to unnecessary healthcare expenditures and patient harm. Despite significant advancements in medical knowledge and technology, prescribing practices often fail to align with evidence-based guidelines or may be founded on biased or incomplete evidence, leading to the overuse, underuse, or misuse of medications (9). This problem is exacerbated in the context of geriatric care, where older adults are disproportionately affected by polypharmacy, adverse drug reactions, and inappropriate prescribing practices. As populations continue to age globally, the burden of ineffective and potentially harmful medications on healthcare systems and individual patients becomes increasingly pronounced (10, 11).

Optimizing prescribing practices is therefore paramount not only for improving patient outcomes but also for mitigating the substantial economic and societal costs associated with futile biomedical research and practices (12). By ensuring that medications are prescribed judiciously and in accordance with the best and holistic available evidence, healthcare providers can minimize waste, enhance the efficiency of healthcare delivery, and ultimately improve the quality of care for older adults. In this context, understanding and addressing the factors contributing to suboptimal prescribing practices, such as age bias, pharmaceutical influence, and inadequate clinician training, are essential steps toward realizing the potential of biomedical research to positively impact patient health and well-being.

The historical perception of osteoporosis can be understood within the broader frameworks of medicalization, pharmaceuticalization, and standardization. Medicalization, defined as the process by which nonmedical problems become defined and treated as medical problems (4), has played a significant role in shaping societal attitudes toward osteoporosis. Once viewed as a natural part of aging, osteoporosis has increasingly been medicalized, with pharmaceutical companies promoting it as a serious disease requiring healthcare intervention (1, 5). However, this medicalization has not been without controversy, with critics accusing the pharmaceutical industry of disease mongering by exaggerating the societal burden of osteoporosis and touting the efficacy of drugs in a misleading manner (13, 14). Questions were raised regarding the true quality of life benefits of the treatments: osteoporosis management is often presented as a cure, when the condition is in fact asymptomatic. Osteoporosis only becomes a medical concern when it translates into fragility fractures, usually following a fall. While fractures are a true concern for the health of postmenopausal women and older adults, the management of osteoporosis can enter the realm of therapeutic futility. While evidence suggests that pharmaceutical treatments may lead to increased bone mass and prevent fractures in the contexts of clinical trials, their real-world efficacy in diverse populations raises questions. The long-term side effects of these treatments combined to their costs enhances these questions. The prevention of fractures in older adults is a complex, multifaceted and multicausal reality.

Pharmaceuticalization refers to the increasing use of pharmaceutical products for the management of conditions perceived as diseases (6, 15). Osteoporosis has undergone intense pharmaceuticalization since the 1990s, with rapid therapeutic expansion and the introduction of several drug classes (16). However, the appropriateness of pharmaceutical interventions for osteoporosis has been questioned, especially given the availability of non-pharmaceutical alternatives such as exercise and dietary adjustments (17). This has led to discussions about de-pharmaceuticalization, or the waning of pharmaceutical products in the management of medical conditions, including osteoporosis. Medicalization complementarily affects the pharmaceuticalization of osteoporosis: the appearance of the osteoporotic issue calls for a public health response, which essentially focuses on pharmaceutical approaches, as they are the only solutions, which fulfill the requirements of healthcare standardization. These feedback loops unfairly and inappropriately promote the pharmaceutical matrix of understanding of the health crisis.

Standardization, meanwhile, has influenced prescribing trends through the development of evidence-based medicine and healthcare policies (18, 19). Clinical guidelines aim to standardize healthcare delivery by encouraging identical behavior among practitioners based on current best evidence. However, standardization is not without biases and societal influences, as healthcare policy reflects the *zeitgeist* of a particular time and place (20). Changes in healthcare policies, resource distribution, and institutional environments can all impact prescribing trends for osteoporosis medication. Overall, understanding the historical perception of osteoporosis and its pharmaceuticalization requires examining the interplay of medicalization, pharmaceuticalization, and standardization within the broader context of healthcare policy and societal attitudes toward aging and disease.

**FIGURE 1:**
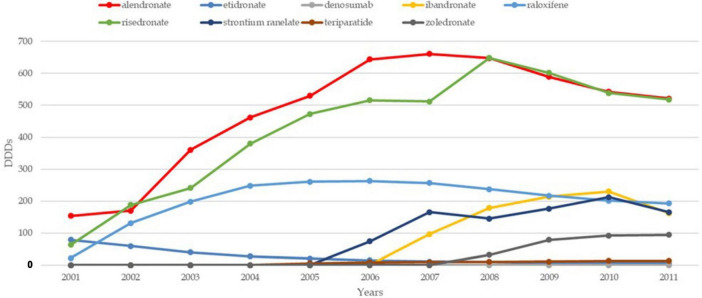
Trends and patterns of osteoporosis drug use in France reported from data aggregated from Hernlund et al. (21). DDD, defined daily dosage.

**FIGURE 2:**
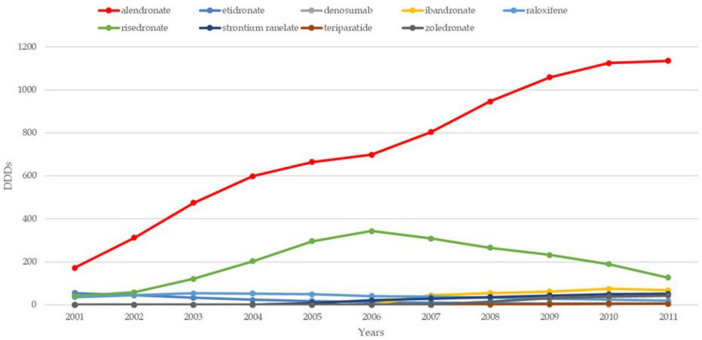
Trends and patterns of osteoporosis drug use in the UK reported from data aggregated from Hernlund et al. (21). DDD, defined daily dosage.

**FIGURE 3:**
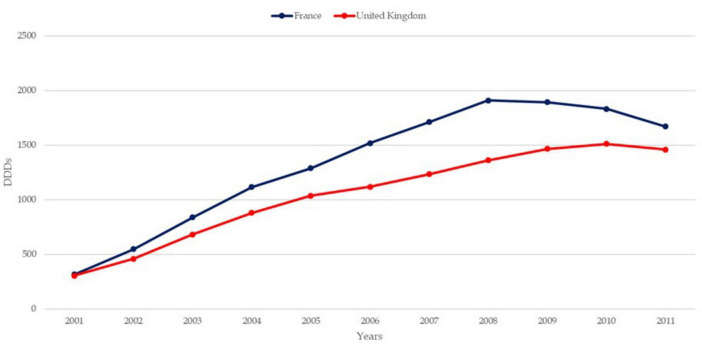
Trends of osteoporosis drug use in France and the United Kingdom from data aggregated from Hernlund et al. (21). DDD, defined daily dosage.

The trends in osteoporosis drug prescribing reveal three distinct prescribing phases: a moderate increase from the early 1990s to 2001 in England, followed by a vigorous increase from 2001 to 2008–2010 in both England and France, and finally, a period of stagnation and contraction, starting around 2008–2010, particularly pronounced in France (21). Evidence confirms the contraction to 2024 in both countries. While England saw a quasi-monopoly of alendronate, France displayed greater diversity in drug use, with bisphosphonates dominating. Data confirm a decline in prescribing trends in both countries, except for denosumab, which increased significantly but remained secondary to bisphosphonates (22, 23). In England, prescriptions declined by 18% for risedronate and 25% for alendronate, while in France, the average decline per molecule was 33% (24). Figures 1–3 illustrate trends and patterns of osteoporosis drug prescription from 2001 to 2011.

Pharmaceutical patents have shaped prescribing trends by incentivizing the promotion of patented drugs through monopolies and aggressive marketing, often at the expense of equally effective alternatives. This dynamic underscores the economic influence on treatment practices and raises questions about prioritization based on profitability rather than patient outcomes. As patents expire, shifts in prescribing behavior highlight the market-driven nature of medical recommendations. Health technology assessments (HTAs), which aim to evaluate drug efficacy and cost-effectiveness, may also perpetuate biases when driven by commercial interests or constrained by focusing on surrogate endpoints like bone mineral density (BMD). While such measures offer quantifiable data, they do not always translate to real-world benefits like reduced fractures, mortality, or improved quality of life. Recommendations centered on such endpoints risk therapeutic futility, offering limited tangible gains to patients. Ultimately, the focus on osteoporosis interventions must emphasize clinically meaningful outcomes, such as reduced fractures, improved mobility, and enhanced well-being. Treatments that fail to deliver these benefits, despite promising metrics in trials, may unjustifiably burden patients without significantly improving their quality of life.

The literature offers multifaceted explanations for shifts in prescribing behaviors regarding osteoporosis. Clinical evidence regarding efficacy and safety profiles of treatments plays a crucial role, as seen in the decline of hormone replacement therapy (HRT) following concerns about its link to breast cancer (25). However, non-clinical factors also shape prescribing trends. Factors such as the availability of diagnostic tools like DEXA scans, their costs, and associated waiting times influence prescription rates (26). Concerns about side effects, changing interpretations of fragility fractures, and broader societal attitudes toward aging and treatment efficacy contribute to the complex landscape of osteoporosis drug prescribing (27). Moreover, issues of trust, fueled by negative publicity and perceptions of pharmaceutical industry practices, have emerged as significant determinants of prescribing patterns. As a response to these challenges, researchers emphasize the importance of improved communication strategies and the exploration of non-pharmacological alternatives for osteoporosis prevention and treatment.

The study investigates trends in osteoporosis drug prescribing and their implications for biomedical research efficiency. By analyzing prescribing patterns, we seek to understand how medicalization, pharmaceuticalization, and standardization influence the use of pharmaceutical interventions for osteoporosis management. Additionally, we explore the potential impact of these trends on healthcare resource allocation and patient outcomes. Ultimately, this research contributes to a better understanding of the efficiency and effectiveness of biomedical research in addressing the healthcare needs of aging populations, particularly in the context of osteoporosis management.

## 2 Materials and methods

To fulfill the study aims, which falls into the field of drug utilization research, the study integrates a double approach: a documentary analysis and semi-structured interviews. Drug utilization research examines how medications are prescribed, dispensed, and used in a specific population, often focusing on patterns, appropriateness, costs, and outcomes. It typically supports evidence-based policies, improved health outcomes, and the rational use of medicines, ultimately aiding in public health interventions and policy adjustments (28). By integrating these two methodological approaches, our study offers a multi-faceted examination of prescribing trends, healthcare policies, and clinical decision-making processes in osteoporosis management. This comprehensive framework is essential for informing strategies to improve patient care and optimize healthcare resource allocation in the context of an aging society.

### 2.1 Documentary data analysis

The documentary analysis component aims to identify patterns and trends in regulatory policies, including marketing authorizations and therapeutic indications in England and France, which directly influence prescribing practices. By delving into the realms of medicalization, pharmaceuticalization, and standardization, we seek to elucidate how these factors shape the utilization of pharmaceutical interventions for osteoporosis. Documentary analysis, as defined in this study, involves a “detailed examination of documents produced across a wide range of social practices taking a variety of forms from the written word to the visual image” (29). Specifically, the analysis focused on identifying patterns and trends within a selected set of policies related to osteoporosis regulation. Policies, in this context, refer to sets of ideas or plans serving as the basis for decision-making in healthcare.

The scope of policy analysis to regulatory policies was limited to marketing authorizations, therapeutic indications, clinical guidelines, and incentive policies. Additionally, the definition of osteoporosis was restricted to adult populations, excluding pediatric and cancer-related osteoporosis. The analysis focused on eight commonly used osteoporosis medications (etidronate, alendronate, raloxifene, risedronate, strontium ranelate, zoledronate, ibandronate, and denosumab). Policy documents were identified through relevant institutions in each country, including supranational organizations like the European Medicine Agency (EMA) and national agencies like the French National Agency for Medicines and Health Products Safety (ANSM) and the British Medicines and Healthcare products Regulatory Agency (MHRA). Documents were extracted and organized using Excel^®^, and when necessary, graphs were created to visualize policy development over time (24).

The analysis primarily focused on identifying trends and patterns in policy development related to outcomes such as marketing authorizations, therapeutic indications, clinical guidelines, and incentive policies. Policies were interpreted based on their intended effects on osteoporosis drug prescribing trends, with positive or negative effects indicated by color-coded figures. To enhance pattern identification, graphic representation strategies were employed to visually interpret trends and regulatory shifts across osteoporosis policies (30). By creating time series graphs and color-coded matrices, we illustrated changes in marketing authorizations, therapeutic indications, and policy updates over time, making patterns in regulatory emphasis and frequency more apparent across both countries. These visual tools allowed for clearer identification of peaks in policy activity, variations in drug approvals, and shifts in clinical guidelines, providing an intuitive and systematic means to analyze regulatory evolution. Such graphical methods facilitated a robust comparative analysis between England and France, highlighting trends and gaps in osteoporosis management policies.

Additionally, a critical assessment of policy documents was included to address any discrepancies or lack of evidence supporting decision-making. Overall, the documentary analysis provided a comprehensive understanding of the policy framework surrounding osteoporosis management in England and France, shedding light on factors influencing prescribing trends and patterns within these healthcare systems.

### 2.2 Interviews with physicians

Complementing the documentary analysis, semi-structured interviews with physicians provide invaluable insights into prescriber decision-making processes. Our interviews dive into various aspects of osteoporosis management, including treatment initiation, medication views, and compliance management, to capture the complexities of clinical practice. Through this qualitative approach, we aim to explore the factors influencing healthcare resource allocation and patient outcomes, thereby enriching our understanding of biomedical research efficiency in addressing the healthcare needs of aging populations. The qualitative analysis of semi-structured interviews involved engaging physicians in both countries through in-depth interviews to explore their perspectives and experiences. Semi-structured interviews, characterized by a discussion guide to ensure key topics are covered while allowing flexibility for exploration, were chosen to delve into the complexity of decision-making processes.

Physicians involved in the diagnosis and management of osteoporosis, including general practitioners, rheumatologists, and geriatricians, were targeted for recruitment. We note that there is little to no practical difference between general practice and family practice in both countries and remark that the management of osteoporosis is more likely linked to primary care in England than in France where it is more frequently linked to specialized care. Gynecologists were excluded from the target population in England due to their limited involvement in osteoporosis management compared to their French counterparts. Hospital pharmacists and radiologists were also included to gather insights into prescribing behaviors. Recruitment of physicians posed challenges due to factors such as time constraints and reluctance to participate (31). Convenience sampling was initially used, leveraging personal connections and gatekeepers within the researcher’s network to facilitate introductions (32). Subsequent recruitment utilized a snowballing strategy, where initial participants referred additional contacts, supplemented by outreach through online registries. A total of 35 interviews were conducted, evenly split between practitioners in France and England. Face-to-face interviews were preferred over phone interviews whenever feasible to foster rapport and ensure optimal data collection. Interviews were conducted in the language appropriate to the respondent’s country of practice, with audio recordings made with consent for transcription purposes. Transcripts were then analyzed using NVivo^®^, employing a generic inductive approach to identify themes and patterns (33).

Primary coding focused on key topics such as costs, diagnosis behavior, drugs, external actors, and prescribing behavior. Queries were conducted to examine patterns and exceptions across demographic characteristics and primary codes. To allow the emergence of the category and subcategory identification, the analysis involved an iterative coding process where initial themes were refined through a systematic review of coded transcripts, ensuring alignment with the study’s research questions. NVivo^®^ facilitated this process by allowing frequency analysis and visual mapping to verify theme prevalence and relationships. Subcategories emerged through focused coding rounds, where we organized broad themes into more specific dimensions, within the overarching theme of medication views. This iterative refinement helped clarify nuanced insights across demographic variables, enhancing the validity of identified patterns. Findings were reported through relevant extracts translated into English, with abbreviations used to denote respondents’ country of practice and medical specialty for clarity. Ethical considerations were paramount throughout the process, with informed consent obtained from all participants, confidentiality maintained, and no financial incentives offered. King’s College London’s Health and Medicine Research Ethics Panel (GSSHM REP) approved the conduct of this study on October 31st 2013.

## 3 Results

### 3.1 Policy analysis

The policy document search yielded a total of 157 documents, between 2015 and 2018. Of these, 70 pertained strictly to England, 80 to France, and seven were European policy documents relevant to both countries. Thirty-two documents related to marketing authorizations, with 13 for England, 12 for France, and seven issued by the EMA. For therapeutic indications, 53 documents were identified, with 32 for England and 21 for France. Clinical guidelines yielded 25 documents, with 17 for England and eight for France, including official guidelines from RCP or NICE in England and AFSSAPS or HAS in France, as well as non-official guidelines from NOGG and GRIO. Incentive policies were only found for England, with eight documents under the QOF.

#### 3.1.1 Marketing authorization

**FIGURE 4:**
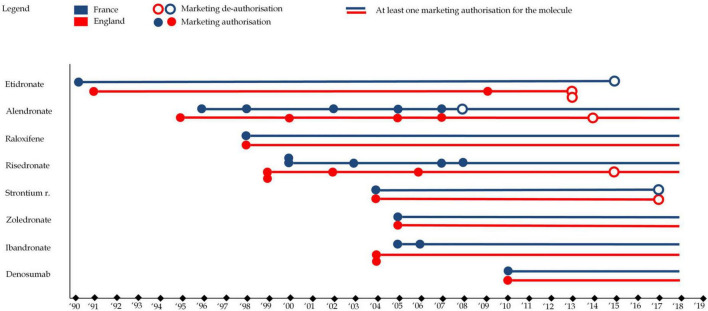
This figure represents drug marketing authorizations for each of the eight molecules in England (in red) and France (in blue). A full circle represents a marketing authorization (any drug format) while an empty circle represents a market de-authorization (any drug format), e.g., the first format of alendronate as authorized in France in 1996 while one format was de-authorized (alendronate 5 mg) in 2008 (though other formats remained on the market).

The analysis of marketing authorizations revealed distinct clusters and outliers, providing insights into the evolution of osteoporosis drug availability in England and France. Notably, etidronate, the first bisphosphonate class drug, obtained marketing authorization in 1990 and 1993 in France and England, respectively. Figure 4 illustrates the evolution of market authorizations in both countries. Overall, the analysis highlights mainly two distinct phases of market activity, with periods of new drug introductions followed by withdrawals, shaping the landscape of osteoporosis treatment availability in both countries.

#### 3.1.2 Therapeutic indications

Therapeutic indications for osteoporosis medications largely align with the patterns observed in marketing authorizations. From 1996 to 2002, the initial therapeutic indications primarily targeted postmenopausal osteoporosis and corticosteroid-induced osteoporosis. This early wave of indications focused on the more traditional approach to osteoporotic fractures, providing a foundation for later developments. The second wave saw an expansion of indications, particularly for postmenopausal osteoporosis, but also included osteoporosis in men. This reflected a broader target population and growing market for osteoporosis treatments.

The evolution of therapeutic indications was marked by market withdrawal due to safety and efficacy concerns. The case of strontium ranelate is particularly instructive. Strontium ranelate was withdrawn in several countries following safety concerns, specifically related to cardiovascular risks. The European Pharmacovigilance Risk Assessment Committee (PRAC) conducted a review of the benefit-risk balance of strontium ranelate, ultimately concluding that the risks associated with the drug, particularly its cardiovascular effects, outweighed the benefits for most patients. The committee’s assessment highlighted the complex risk/benefit balance of the drug, as it had demonstrated efficacy in reducing fractures, yet the potential for serious cardiovascular events, including myocardial infarction and stroke, raised significant concerns. Strontium ranelate’s withdrawal underscores the dynamic nature of market authorization and the importance of ongoing pharmacovigilance in assessing the long-term safety of medications. This process exemplifies how medications may be removed from the market or their indications restricted as new safety data emerges. Such decisions reflect the broader risk/benefit evaluation required in the treatment of osteoporosis, where the potential benefits of fracture prevention must be weighed against the risks of adverse events, especially in vulnerable populations.

**FIGURE 5:**
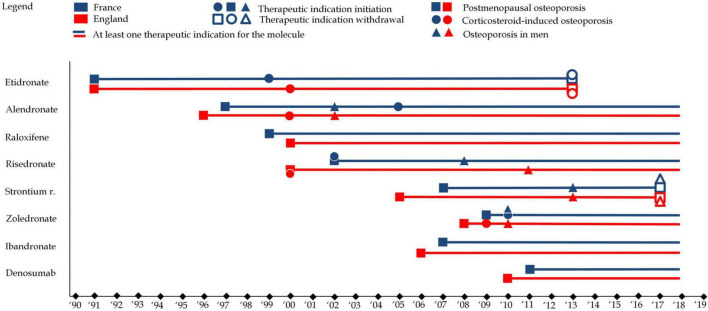
This figure represents therapeutic indications for each of the eight molecules in England (in red) and France (in blue). Squares refer to therapeutic indications associated with postmenopausal osteoporosis, while circles refer therapeutic indications associated with to corticosteroid-induced osteoporosis and triangles refer therapeutic indications associated with to osteoporosis in men. A full shape represents an additional indication) while an empty shape represents the withdrawal of an indication e.g., alendronate received an indication for postmenopausal osteoporosis in 1997 in France, osteoporosis in men in 2002 and corticosteroid-induced osteoporosis in 2005.

**FIGURE 6:**
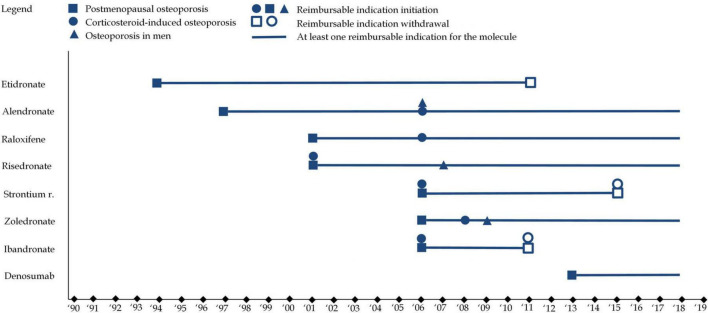
This figure represents French reimbursable therapeutic indications for each of the eight molecules. Squares refer to therapeutic indications associated with postmenopausal osteoporosis, while circles refer therapeutic indications associated with to corticosteroid-induced osteoporosis and triangles refer therapeutic indications associated with to osteoporosis in men. A full shape represents an additional indication) while an empty shape represents the withdrawal of an indication.

While all osteoporosis drugs received an indication for postmenopausal osteoporosis, variations existed in the specificity of indications. For instance, etidronate and ibandronate were limited to vertebral osteoporosis, whereas other medications, like alendronate and zoledronate, had broader indications covering both vertebral and femoral osteoporosis. These distinctions, along with market withdrawals, have shaped the therapeutic landscape for osteoporosis, reflecting both regulatory caution and evolving scientific understanding. Figure 5 illustrates the evolution of therapeutic indications. Figure 6 represents reimbursable therapeutic indications in France.

#### 3.1.3 Clinical guidelines

Clinical guidelines are essential for standardizing medical practice, offering evidence-based recommendations for the diagnosis, treatment, and prevention of conditions like osteoporosis. Given that early intervention in osteoporosis can prevent fractures and improve quality of life, the evolution of these guidelines has profound implications for healthcare delivery. This section focuses on the development of osteoporosis guidelines in France and England, emphasizing their role in shaping prescribing practices and highlighting the tension between evidence-based medicine and the potential for futile treatment.

Osteoporosis clinical guidelines in England and France began to take shape in the late 1990s and early 2000s. Initially focused on postmenopausal osteoporosis, they gradually expanded to include other types such as corticosteroid-induced and male osteoporosis. Throughout their evolution, medications like alendronate, risedronate, and etidronate were commonly recommended. However, as the guidelines evolved, certain treatments gained prominence, while others—such as raloxifene—saw a decline in favor due to shifts in evidence and focus on specific patient populations. In England, alendronate became a first-line treatment for postmenopausal osteoporosis, reflecting its strong evidence base. Meanwhile, in France, guidelines maintained a broader array of first-line options, offering more flexibility in treatment choices.

The role of corticosteroid-induced osteoporosis in clinical guidelines, once an under-recognized condition, was notably emphasized in the early 2000s, prompting recommendations for medications like alendronate and risedronate. However, this shift was not universal across countries, and differing policy priorities and the availability of evidence led to variations in treatment recommendations. Similarly, osteoporosis in men, though increasingly acknowledged, was still treated with less consensus, signaling the need for further research.

One critical trend in the evolution of these guidelines is the pharmaceuticalization of osteoporosis management. Medications, such as bisphosphonates, gained prominence, often at the expense of preventive measures like exercise and nutrition, which were less emphasized despite their role in bone health. This reliance on pharmaceutical treatments reflects a broader tendency in clinical guidelines to prioritize medicalized, drug-centric approaches to disease management, sometimes overshadowing alternative interventions.

The withdrawal of certain medications, such as strontium ranelate, further complicates the landscape, underscoring the role of market dynamics and regulatory decisions in shaping clinical recommendations. These shifts reflect the evolving understanding of treatment efficacy and safety but also highlight the potential for futile treatments to persist due to vested interests and the slow pace of evidence accumulation.

**FIGURE 7:**
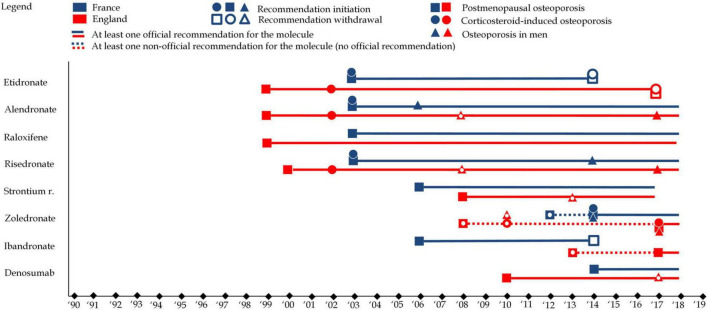
This figure represents clinical guidelines for each of the eight molecules in England (in red) and France (in blue). Squares refer to clinical guidelines associated with postmenopausal osteoporosis, while circles refer clinical guidelines associated with to corticosteroid-induced osteoporosis and triangles refer clinical guidelines associated with to osteoporosis in men. A full shape represents an additional clinical guideline while an empty shape represents the withdrawal of a clinical guidelines.

Ultimately, while clinical guidelines are meant to standardize care and optimize outcomes, they must be critically evaluated to avoid perpetuating futile treatment strategies. By focusing on evidence that genuinely improves patient outcomes and reconsidering the role of pharmaceuticals in osteoporosis management, guidelines can help shift the focus toward more sustainable and effective approaches to treatment. However, evidence-based clinical guidelines are fundamentally limited by available evidence. As evidence generation is a complex and expensive process, guidelines are subject to funding bias. Figure 7 represents changes in clinical guidelines in both countries.

#### 3.1.4 Incentive policies

Incentive policies play a crucial role in shaping healthcare practices, and their impact on osteoporosis management in England is notable, as no such policy exists in France. The Quality and Outcomes Framework (QOF), established in 2004 as part of the General Medical Services contract for General Practitioners (GPs) in the NHS, serves as a performance management and remuneration system. While the QOF aims to align clinical practices with official recommendations and drive behavioral change, its influence on osteoporosis management is somewhat limited. Osteoporosis was first included as an entry in the QOF in 2012–2013, but it ranks low in priority compared to other conditions like diabetes or hypertension. With only six to seven points associated with osteoporosis objectives, it falls far behind in incentivization. Nonetheless, the QOF outlines three main objectives for osteoporosis management. Patient Registration: GPs are encouraged to include osteoporotic patients in a register, particularly those aged 50–74 with a fragility fracture and DEXA-confirmed osteoporosis, as well as those aged 75 and older with a fragility fracture. Treatment Rates: GPs are incentivized to ensure that between 30 and 60% of eligible patients receive treatment for osteoporosis, based on age and fracture history. Treatment Thresholds: There are age-specific treatment thresholds, with stricter criteria for patients younger than 75 compared to those aged 75 and older.

Notably, the QOF objectives do not address corticosteroid-induced osteoporosis or osteoporosis in men. Moreover, patients with diagnosed osteoporosis but without a fracture are not considered a target population. The percentage range for treatment rates acts both as an incentive and a deterrent, with lower and upper thresholds encouraging GPs to treat a specific proportion of eligible patients. Overall, the incentive policies within the QOF provide some framework for osteoporosis management in England. However, they exhibit limitations in addressing certain patient demographics and types of osteoporosis, suggesting room for refinement and expansion to enhance the effectiveness of osteoporosis care within the NHS.

#### 3.1.5 Overall policy analysis

**FIGURE 8:**
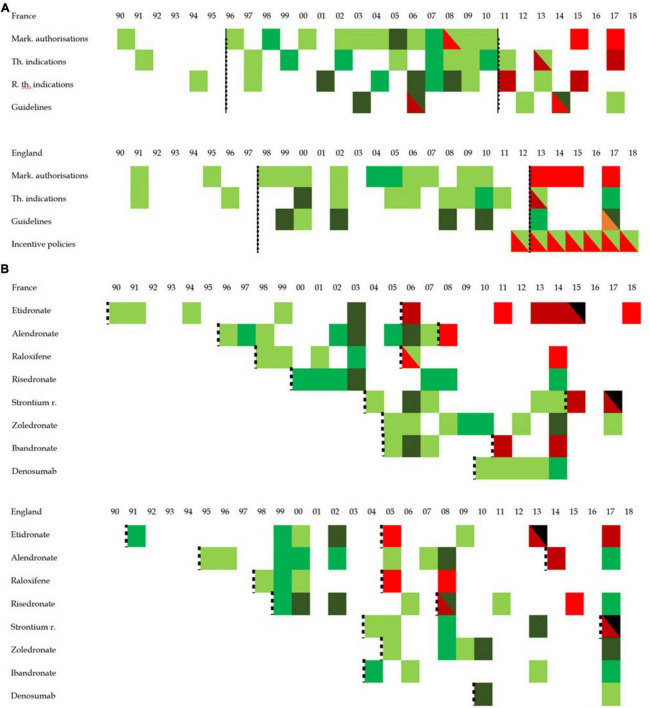
Graphic overview of policy development in England and France. This figure represents the concentration of positive or negative policy issued between 1990 and 2018. Positive policy refers to a policy interpreted as opening the way to more prescription while a negative policy is one that is interpreted as narrowing the possibility for prescription. While white cells represent the absence of any policy, the darker the green the more positive policy toward the prescription of osteoporosis pharmaceutical were published, and the darker the red, the more negative policies discouraging the prescriptions of osteoporosis pharmaceutical products were issued. The black dotted lines group periods into clusters. **(A)** Represents the policy overview by policy type while panel **(B)** looks at the policy overview from the standpoint of molecules. In panel **(B)**, black cells refer to the molecule market withdrawal. While the dotted line was applied for the overall policies in panel **(A)** (thereby creating period for overall prescribing trends), panel **(B)** proposes periods per molecule in an attempt to investigate drug patterns.

Figures 8A, B provide an overall view at the policy landscape in both countries from four perspectives, calendar outlook (evolution over time), binational comparison (France vs. England), policy type outlook (analysis by policy type) and a molecule outlook (analysis by molecule). It reveals several elements contributing to understanding prescribing trends and patterns for osteoporosis drugs in both countries. First, the phases associated with therapeutic expansion from the early 1990s to the late 2000s or early 2010s are associated with policies supporting that expansion. Whether such policy framework in both countries caused or accompanied the trend, this policy analysis cannot say. However, policies could, had they not occurred, have prevented this increase. This is the case of marketing authorizations and (reimbursable) therapeutic indications policies. Thus, while it cannot be said that these policies generated the therapeutic expansion, it can be safely said, from these findings, that they enabled and contributed to it. Second, the policies largely explain the differences of prescribing patterns of osteoporosis drugs, in particular the clinical guidelines. By ranking molecules and imposing treatment lines, English guidelines have *de facto* enabled and created the near monopoly of alendronate, while, by pooling most molecules in first line in France, French authorities have left the decision to physicians. This can explain the more diverse treatment patterns observed in France. Within the sociological medical framework, we interpret policies related to osteoporosis from the early 1990s to 2010 as promoting and contributing to the medicalization and pharmaceuticalization of osteoporosis. By standardizing care of osteoporosis toward increased diagnosis (e.g., incentive policies in England) and providing diagnostic thresholds and behavioral responses including pharmaceutical products, these policies have encouraged the use of pharmaceutical products for the treatment of osteoporosis.

Similarly, policy changes seem to contribute or accelerate the phenomenon of prescribing stagnation and contraction, more than it initiated it. With prescribing stagnation starting in France in 2008 and in England in 2010, the data shows the first policies expected to have a negative impact on prescribing 2 years after the first signs of prescribing changes occurred. This suggests that the policy framework contributed to a de-medicalization or de-pharmaceuticalization of osteoporosis. First the withdrawal of etidronate and strontium ranelate (and ibandronate in France) limited the therapeutic arsenal available to physicians. The near absence of any new policy supporting the continuation of the pharmaceutical process may be more significant.

### 3.2 Insights of interviews

#### 3.2.1 General results

We conducted 35 interviews in 2014 and 2015, all of which were individual interviews, except for one, which included two respondents. On average interviews lasted 41 min. While most interviews were conducted face-to-face, eight were telephone interviews. Half of the interviewees practiced in France (*n* = 17) with the other half practicing in England (*n* = 18). In France, all interviewees practiced in the north-western part of the country within a 200 km radius from the city of Nantes, Pays de la Loire. In England, most interviewees practiced in the Greater London area and three interviewees practiced in Oxfordshire and one in Cumbria. Eight respondents were geriatricians, 12 were GPs, eight were rheumatologists, two were gynecologists, two were radiologists and three were hospital pharmacists. Regarding durations of practice, 14 respondents had practiced for more than 20 years, seven for 11–19 years and 14 for 10 years or less. Nineteen respondents were male and 16 were female. Eighteen respondents practiced in a hospital, of which eight were in a geriatric ward and 16 worked in private practices. Twenty respondents reported having urban patients essentially, nine a mix of both urban and rural patients and six with rural patients essentially.

#### 3.2.2 Management of osteoporosis

Physicians’ approaches to osteoporosis management exhibit both uniformity and diversity, influenced by factors such as country, medical specialty, and patient characteristics. In England, a systematic approach to the decision to screen prevails, largely driven by adherence to official (NICE) and non-official (NOGG) guidelines. The FRAX – a tool to “evaluate fracture risk of patients” (34) – plays a central role in this decision-making process, as illustrated by the consistent use reported by most interviewed physicians.

EN_GP3: “So, they’ll take a weekly alendronic acid [alendronate] and a daily calcium plus vitamin D. And that decision is based on either a DEXA scan which shows the osteoporosis or doing a FRAX score. And that’s the end of the interview, isn’t it? It’s just such a simplistic decision with me. Surely there isn’t more to it than that!”

However, discrepancies exist, with some practitioners expressing skepticism or underuse of the FRAX score, citing concerns about its epidemiological rigor or prioritization behind cardiovascular risk assessments.

EN_GP4: “You might sometimes think of doing a FRAX score, but I guess, to be honest, it’s so much secondary to me behind cardiovascular risk but yes, I probably don’t do it as much as I should. But I do it sometimes.”

In contrast, the decision to screen in France appears less standardized, varying significantly across physician specialties. While rheumatologists demonstrate a more systematic approach, many GPs either do not use or are unaware of the FRAX tool. Referral patterns also differ, with frequent or systematic referrals to rheumatologists observed in France, offering a pathway for osteoporosis assessment beyond primary care.

Interviewer: For example, do you use the FRAX? It is a tool, a questionnaire. FR_GY1: No. Rheumatologists do use it a lot though.

Interviewer: The tool FRAX, have you heard about it? FR_GP3: FRAX? Interviewer: Yes, F – R – A – X [spells]. FR_GP3: So, no, I don’t believe so. I don’t believe so… I don’t believe so. I’d say I’ve seen that once, on a DEXA scan report. I don’t remember anymore. FRAX. I should take note. I don’t recall.

FR_RH4: We may use FRAX, the FRAX score, F-R-A-X [spells], to determine the probability of major or hip fractures at 10 years. Although it is not perfect, it can point out, encourage us to start a prevention treatment.

Despite differences in the decision to screen, the screening process itself largely revolves around DEXA scans in both countries. However, nuances exist, particularly in France, where selective screening is more common among GPs, with certain patient populations, such as those receiving corticosteroid therapy or those with fragility fractures, more likely to undergo evaluation. Patient age also influences screening practices, with older patients less likely to be screened routinely, especially in France.

Interviewer: Does it occur to you to conduct them [DEXA scans] on the very old? FR_GP1: No, I don’t think we can say that. It is hard to say, to give you a definitive yes, I’d need to go through my patient files. I don’t think that I have a lot of patients [feminine term used in French] who receive a DEXA scan after the age of 80 years. It takes place earlier, maybe 70 years, possibly 75 years.

Resource availability and reimbursement policies further shape the screening process, with access to DEXA scanners and reimbursement criteria affecting the frequency and timing of screenings. The reflex to refer patients to specialists, particularly rheumatologists, is more prevalent in France, where the availability of DEXA scans and access to specialists may vary regionally.

Interviewer: You say that when you do a DEXA scan you order an appointment for the patient in rheumatology? FR_GP7: In general, yes. It has not happened recently, but this is what I’d do. I’d go see the DEXA results and then I’d seek the advice from a rheumatologist.

While the screening process for osteoporosis diagnosis primarily relies on DEXA scans, the decision to screen exhibits considerable variability, influenced by factors such as guidelines adherence, specialty practices, patient demographics, and resource availability. Standardization is more evident in England, where the FRAX tool guides decision-making across specialties, whereas France presents a more heterogeneous landscape, with referral patterns and screening practices varying widely among physicians.

The decision to treat osteoporosis equally involves a complex interplay of factors, including physician perception, patient preferences, guidelines, and reimbursement considerations. Physicians generally perceive treating osteoporosis as the default choice upon diagnosis. However, probing deeper reveals instances where not treating is considered, often due to contraindications such as advanced age or renal impairment. The decision not to treat is also influenced by patient preferences, with some patients declining treatment, particularly in France where patient autonomy in medical decisions appears more significant in the decision-making process.

Interviewer: Are there certain conditions where a patient with risk factors and a positive DEXA scan would be associated with a decision not-to-treat? FR_RH1: No. [silence] Interviewer: Not any case? FR_RH1: Hm, except if there are important contraindications. Interviewer: Is there for example an age limit which would make you doubt of…? FR_RH1: Yes, well, age limit… In fact, obviously, after 80 years we ask to be a bit prudent with treatments because there are renal impairments, things like that.

EN_RH1: In a very holistic way it is discussed with the patient. Patient is given a choice, we never force patients to be commenced, to be… or at least we try to be. We are an advisory role rather than dictating what should be done.

Polypharmacy, while recognized as a concern, is not a significant barrier to prescribing osteoporosis medications. Physicians prioritize patient acceptability and compliance over potential drug interactions. Osteoporosis is rarely viewed as a secondary condition, though treatment decisions may be influenced by competing health priorities, such as polypharmacy or patient age.

*FR_RH2: I am thinking, I am thinking… So yes, it can happen [that I do not prescribe] in the context of renal insufficiency. It can be a barrier to treatment but otherwise there aren’t many barriers. The main barrier, that’s renal insufficiency.*

*Interviewer: Can polypharmacy make you doubt the need to prescribe?*

*FR_RH2: Not really, no, it isn’t really a bother to us. Mainly because we have relatively simple treatments. Two pills per month, an infusion once a year, a subcutaneous injection every 6 months, it remains… It is not something very burdensome*, so it is not a factor that I am interested in.

Physicians in England predominantly prescribe alendronate, with few deviations from this choice. This trend is partly attributed to historical prescribing incentives and familiarity. Denosumab is emerging as an alternative, especially in settings where practical considerations favor its administration over bisphosphonates.

Interviewer: You mentioned that the first course of treatment is usually oral bisphosphonate? EN_RH2: In terms of the patients that come to us, the first course of treatment for any patient is lifestyle measures. You know, weight-bearing exercises, fall prevention, physiotherapy etc. And then of course there’s assessment of vitamin D and calcium in dietary intake.

EN_GP5: I can’t think of the last time I prescribed something else [from alendronate]. Because the prescribing incentive scheme around alendronate was quite a few years ago, so you know how long my memory lasts.

In contrast, French physicians exhibit greater diversity in treatment selection, with bisphosphonates, raloxifene, and denosumab among the commonly prescribed options. Treatment choices are influenced by factors such as patient age, location of osteoporosis, and recommendations from specialists. Collaboration with other specialists, such as rheumatologists or gynecologists, often guides treatment initiation among French general practitioners.

Interviewer: And regarding treatments, how do you proceed? FR_RH1: So, how do I proceed? Hm, if the woman is younger than 65 and does not have breast cancer with treatment contraindications, I put raloxifene as first intention if osteoporosis is predominantly located at the lumbar spine. You know, we measure at two sites, lumbar spine and neck of the hip. So, here, if it is at the spine, it will be more raloxifene then if it is really very low at the level of the neck of the hip, bisphosphonates as first intention must be privileged.

Physicians in both countries are aware of treatment reimbursement considerations, which influence prescribing behaviors. While English physicians commonly refer to NICE guidelines for treatment decisions, French physicians infrequently mention HAS guidelines. Reimbursement status plays a significant role in treatment selection, with physicians opting for reimbursed medications to ease financial burden on patients.

The decision to treat osteoporosis is multifaceted, influenced by physician perceptions, patient preferences, and healthcare system factors. While prescribing patterns differ between countries, a common thread is the importance of patient-centered care and adherence to reimbursement policies. Understanding these dynamics is crucial for optimizing osteoporosis management and improving patient outcomes.

#### 3.2.3 Attitudes toward patient age

The analysis of physician attitudes toward patient age in the context of osteoporosis drug prescribing reveals a complex dynamic between chronological age and physiological age. Chronological age, defined simply as the number of years lived, emerges as a significant factor in the decision-making process for some physicians, while others emphasize the importance of physiological health status over age alone. Numerous interviewees acknowledged the role of chronological age in their decision-making process regarding osteoporosis diagnosis and treatment. However, the extent to which it influences their decisions varies considerably among practitioners. For many, chronological age does not significantly impact their medical decisions, as exemplified by statements such as, “Age is not a factor to make a decision” (EN_RH1). These physicians maintain that they treat patients based on their overall health rather than their age.

Conversely, some physicians express a tendency toward diminished investigation and treatment of osteoporosis in older age groups, particularly those aged 80 and above. One French GP mentioned being less inclined to search for osteoporosis in individuals over 80 due to the presence of other health issues and polypharmacy (FR_GP4). Similarly, a French rheumatologist highlighted that patients over 80 may be systematically excluded from osteoporosis investigation and treatment if they are deemed not well enough to justify management or if they are considered sufficiently well that osteoporosis is not a primary concern (FR_RH4).

Several physicians across different specialties and countries acknowledged using age thresholds as criteria for treatment decision-making. For instance, an English GP mentioned considering age thresholds around 75 and 80 when assessing the balance between benefits and side effects of treatment (EN_GP1). Similarly, another English geriatrician indicated that individuals over 80 with low-impact fragility fractures would be automatically considered for treatment (EN_GE3).

One notable finding is the potential confusion between chronological age and physiological age, where physicians assume a shorter life expectancy based solely on age. This assumption may lead to the exclusion of patients with a younger physiological age from osteoporosis investigation and treatment. Some practitioners mentioned using chronological age as a proxy for physiological age, without necessarily considering the individual’s overall health status (FR_GP1). Overall, the use of chronological age in osteoporosis management appears to be plural and multidirectional. While some physicians may systematically exclude older patients from treatment, others may automatically diagnose and treat osteoporosis based on age alone.

Interviewer: So, if someone is healthy at 95, you…EN_RH1: Oh, of course! I’ve got plenty of such ladies. I mean, if they don’t have medical contraindications. Age is not […] a factor to make a decision.

FR_GP4: I’d say that there is the category of around 60, 70 years, with whom we will conduct diagnoses, we will try to treat. That’s certain that after 80 years, we might be less inclined to search for osteoporosis. On the other hand, we will do prescriptions.

Interviewer: So, regarding age, you think that beyond which threshold will you stop questioning [the need for] a DEXA scan?FR_GP5: Oh 90, certain. After 80 years, I start questioning myself. […] But it is true that at 90 years, hm, I don’t see [the need] … except if there is history of falls, and even then…

FR_RH1: Yes, well, an age limit… Obviously, after 80 years, we ask to be a bit more prudent with treatments because it is associated with renal impairment, things like this. Now, someone who is 80 and who is very well, who has a physiological age of 70, 75, we see more and more, who has never fractured and who is still active, maybe it is worth treating.

In summary, physician attitudes toward patient age in osteoporosis management vary widely, with some emphasizing chronological age as a significant factor, while others prioritize physiological health status. The complexity of this relationship highlights the nuanced approach to treatment decision-making and the consideration of individual patient characteristics beyond age alone. The analysis of physiological age in the context of osteoporosis management sheds light on several key factors that influence treatment decisions among prescribers. While chronological age may not directly dictate treatment choices, physiological age, which encompasses factors such as renal function, overall health status, and the burden of treatment, emerges as a significant consideration.

One prominent factor influencing treatment selection is renal function, often considered a reflection of physiological age. Poor renal function can act as a barrier to initiating treatment, particularly with bisphosphonates, which are commonly avoided in patients with compromised renal function. This is evident in statements such as, “If they have a shitty kidney, well, bisphosphonates, that’s sorted, they are put in the bin” (FR_GP3). Similarly, English practitioners adjust treatment choices based on renal function, opting for alternatives like risedronate in cases of borderline kidney function (EN_GE5).

Physicians, particularly in France, demonstrate a preference for food supplements over pharmaceutical treatments in physiologically older patients. Vitamin D and calcium supplements are often seen as alternatives to pharmacological interventions, especially in individuals over 80 who may have multiple comorbidities and polypharmacy. This approach reflects a consideration of treatment simplicity and patient compliance, as highlighted by statements like, “When they are very old, with polypharmacy…I am going to insist a lot on vitamin D” (FR_RH2).

The burden of treatment administration also influences decision-making, especially in older patients. Physicians tend to favor less burdensome treatments, such as zoledronate infusions administered annually, particularly in patients with severe polypharmacy or those who may struggle with complex medication regimens. Simplifying treatment regimens is viewed as essential for improving patient compliance and adherence, as noted by statements like, “When they are older, you can’t give them complicated things. You have to simplify to the maximum the treatments” (FR_GP3).

Overall, physiological age plays a critical role in treatment selection, with prescribers considering factors such as renal function, overall health status, and treatment burden. While chronological age may serve as a proxy for physiological age, the emphasis lies on the patient’s ability to tolerate and adhere to treatment regimens. The integration of physiological age considerations into treatment decisions reflects a patient-centered approach aimed at optimizing outcomes and reducing treatment-related burdens. In summary, the analysis of physiological age highlights the multifaceted nature of treatment decision-making in osteoporosis management. By considering factors beyond chronological age, such as renal function, overall health status, and treatment burden, prescribers aim to tailor treatment regimens to individual patient needs, ultimately enhancing treatment efficacy and patient outcomes.

#### 3.2.4 Shifting trust and physician behavior

The theme of trust emerged as a critical factor influencing prescribing patterns, alongside considerations of age, both chronological and physiological.

Physicians’ trust in pharmaceutical products and the pharmaceutical industry significantly impacts prescribing behaviors. In England, there appears to be a relatively high level of trust in pharmaceuticals, particularly bisphosphonates like alendronate. This trust is bolstered by evidence of efficacy and the endorsement of national health authorities like NICE. However, in France, trust in pharmaceuticals seems more nuanced, with physicians expressing varying levels of confidence depending on factors like clinical evidence, past experiences with specific drugs, and perceptions of industry influence. Concerns about safety and efficacy, especially in light of controversies surrounding certain medications, contribute to a decline in trust among some French physicians.

Interviewer: In terms of overall prescription, do you feel that you have been prescribing more over time, or have you started increasing your…EN_RH3: No, I prescribe more and more. So, I think the evidence is, as I’ve practiced, it’s become very clear that, hm, most people benefit from it, for whom it’s indicated. The age is no bar from treatment, hm, and I probably still err on the side of conservatism, I don’t like giving people tablets and I suspect I should push myself to give more.

Interviewer: Do you trust these treatments? Do you believe in their efficacy?FR_RH1: Bisphosphonates, yes, I am confident in these. There are studies with massive samples. We see ourselves the decline in the number of fractures, the improvement of the DEXA results as well as the non-aggravation, because if we didn’t treat… there. Forsteo [teriparatide] works very well, but it is not well accepted, with patients having a hard time receiving injections every day. It is difficult. I’d say the one I have the least trust in, it is Protelos [strontium ranelate] [the interview is interrupted by a phone call for a few seconds]. Yes, Protelos, so, it is because… Well in fact the Laboratoires Servier [note: the manufacturer of strontium ranelate]… Well, we can trust the journalists [note: here the rheumatologist referred to the clinical controversy surrounding this molecule and supposedly occulted cardiovascular risks]. There, Protelos, I’d say is the one I trust the least. Other than that, Prolia [denosumab], I find it very good, it works very well.

FR_GP5: There, I prescribe very little, hm, I think that I don’t believe in them in fact. I am not at all convinced, hm, regarding medications in France… Hm. I have the feeling… extremely doubtful. There were prescriptions like Bonviva [ibandronate] that I had initiated which were later withdrawn from the market. So, it puts me at odds with my patients, hm. I don’t have the time to study all the guidelines and obviously the indications of the new drugs. I trust in the health authorities, but eventually I realize that with hindsight, I shouldn’t trust them because, in the case of Bonviva for the management of osteoporosis, there was an indication and at the end of the day there wasn’t any effect on osteoporosis of the spine or conversely of the hip. This was an element that I had not realized because I thought it was a treatment like Actonel [risedronate], but once a month, that it had exactly the same indication because the health authority had given it the same authorisation. I did not imagine that we could put on the market a drug that did not have all the indications, or at least not the same results of the former drug.

FR_GP6: I don’t prescribe systematically, that’s for sure. What I mean is… Because we had the impression that during a time, osteoporosis was spoken a lot about. We had the impression that we were pressured by the pharmaceutical industry, at the time, saying that osteoporosis concerned all women, that treatment was necessary.

The relationship between physicians and health authorities also plays a crucial role in prescribing trends. In England, the influence of organizations like NICE and CCGs is significant, with physicians often adhering closely to treatment guidelines and recommendations. This reliance on standardized protocols may stem from a sense of trust in the expertise and oversight provided by health authorities. Conversely, in France, physicians enjoy more autonomy in decision-making, leading to a greater diversity of prescribing practices. While this autonomy may foster independence, it can also contribute to uncertainty and confusion, particularly when faced with conflicting information or guidance.

The role of cost containment strategies also intersects with trust dynamics. In England, physicians are cognizant of their role in limiting healthcare spending, often deferring to formulary restrictions and reimbursement criteria set by health authorities. This reflects a trust in the system’s ability to balance cost-effectiveness with patient care. Conversely, in France, physicians may perceive cost considerations as less central to their practice, leading to differing attitudes toward healthcare rationing and resource allocation.

EN_PH2: There’s such a high level of scrutiny over high cost drugs that they’re…that the commissioner is basically putting it through a computer and it spots any anomalies so I know [name] they’ll refuse to pay. So, say a patient’s fridge broke and their teriparatide had to be thrown away, then we would give them more, but they would question why we were giving them more. So, it’s very…there’s no scrutiny, really, of the other drugs as much.

The shifting landscape of trust between physicians, pharmaceutical products, and health authorities has significant implications for prescribing trends and patient care. In England, a more centralized approach to decision-making may streamline processes and ensure consistency in treatment practices. However, this model could also limit individualized care and innovation. In France, greater physician autonomy allows for flexibility and personalized treatment plans but may result in variability and inefficiencies. Understanding these trust dynamics is essential for optimizing osteoporosis management and improving patient outcomes. By fostering trust among healthcare stakeholders and promoting evidence-based practices, policymakers can support more effective and efficient biomedical research, ultimately benefiting aging populations grappling with osteoporosis and other age-related conditions.

## 4 Discussion

The observed decline in osteoporosis drug prescribing reflects broader societal changes and healthcare dynamics. Factors such as changing perceptions of aging, policy influences, and healthcare provider decision-making contribute to these trends. In the context of reducing futile biomedical research, our study emphasizes the importance of understanding prescribing trends to optimize resource allocation and improve patient care. By identifying factors driving the decline in osteoporosis drug prescribing, policymakers and healthcare providers can develop targeted interventions to address barriers to prevention and treatment uptake and enhance patient outcomes. The need to minimize futile biomedical research is evident: our study reveals that prescribing patterns are influenced by a complex interplay of factors, some of which reflect the disproportionate focus on pharmaceutical interventions driven by economic incentives, rather than patient-centered outcomes. These insights highlight the importance of a more holistic approach to osteoporosis management, one that prioritizes preventive measures and non-pharmacological strategies alongside medical treatments. In doing so, we can reduce the resources spent on ineffective treatments and research, while redirecting efforts toward evidence-based practices that offer meaningful benefits to patients’ health and quality of life.

The evolving landscape of osteoporosis drug prescribing highlights the deep interconnections between healthcare policies and broader societal trends of medicalization, pharmaceuticalization, and standardization. Policies surrounding the treatment of osteoporosis often reflect and reinforce a medicalized approach to aging, where aging-related conditions are predominantly framed as diseases requiring pharmaceutical intervention. This shift from preventive or lifestyle-based approaches to pharmaceutical solutions has been influenced by the strong presence of the pharmaceutical industry, which has shaped both public perceptions and clinical guidelines. As medications such as bisphosphonates became widely prescribed, they were not only positioned as the cornerstone of osteoporosis management but also standardized the treatment protocol across healthcare systems. The rapid adoption of these drugs, alongside the expansion of diagnostic categories like osteopenia, exemplifies the pharmaceuticalization of healthcare, where medical products dominate the response to health issues, often overshadowing non-pharmacological interventions. Policy decisions that favor pharmaceutical interventions can thus inadvertently perpetuate a cycle of dependency on medications, often at the expense of broader, more holistic approaches to health promotion. Furthermore, the standardization of treatment protocols through guidelines and health technology assessments, while intended to ensure consistency and quality of care, may also reduce the flexibility of clinical decision-making, narrowing treatment options and potentially overlooking individual patient needs. These policies, while aiming for efficiency and universal applicability, can result in the reinforcement of a pharmaceutical-driven healthcare system, where the market dynamics and vested interests of the pharmaceutical industry heavily influence both treatment options and patient outcomes. Reflecting on these trends, it becomes clear that policy frameworks must be critically evaluated to ensure they do not merely mirror the interests of industry, but rather prioritize patient-centered care that balances medical, social, and preventive strategies for osteoporosis management.

The withdrawal of certain osteoporosis medications from the market, while often framed as a response to safety concerns or lack of efficacy, also warrants a critical interpretation. Such withdrawals reflect a broader market-driven dynamic, where the financial interests of pharmaceutical companies intersect with regulatory oversight. The decision to remove drugs, like strontium ranelate, not only questions their long-term therapeutic value but also highlights the tension between clinical needs and the economic incentives tied to drug development. These withdrawals can serve as a reminder that, while market forces shape prescribing practices, they also reveal the inherent limitations of over-reliance on pharmaceutical solutions, signaling the need for ongoing scrutiny of the benefit-risk balance in treatment guidelines.

Our qualitative analysis revealed the central role of trust in shaping prescribing behaviors, highlighting the need for interventions to rebuild trust in healthcare institutions and pharmaceutical products. Healthcare provider decision-making also plays a crucial role in prescribing patterns. Our findings suggest that trust, both in healthcare institutions and pharmaceutical products, strongly influences physician prescribing behaviors. Addressing trust issues through targeted interventions and education initiatives may help mitigate barriers to effective prevention, treatment uptake and could lead to improved patient outcomes.

The decline in osteoporosis drug prescribing cannot be attributed to a single cause but rather stems from a combination of factors. Changing perceptions of aging and health, influenced by societal attitudes and healthcare policies, play a significant role in shaping prescribing behaviors. Worldwide trends of osteoporosis prescribing trends are plummeting in a similar fashion as in France and England. While I cannot establish based on this study that a similar phenomenon is the result of identical causes, some elements of contextualization can be discussed. While explanations for the increase do not raise controversy, the contraction in England and France remains largely unexplained, as the literature relays (21, 35, 36). This rare case of depharmaceuticalization raises questions regarding the allocation of resources and healthcare planning. Our study highlights the importance of considering these broader contextual factors when interpreting prescribing trends and developing interventions to address them. The findings of our study have important implications for healthcare policy, practice, and research. By understanding the factors driving prescribing trends, policymakers can develop evidence-based interventions to promote appropriate treatment uptake and improve patient outcomes. Healthcare providers can also benefit from insights into patient preferences and concerns, enabling them to tailor treatment plans more effectively. In summary, our study provides valuable insights into trends in osteoporosis drug prescribing and their implications for reducing futile biomedical research. By identifying factors driving prescribing patterns, policymakers and healthcare providers can develop targeted interventions to address barriers to prevention and treatment uptake and enhance patient outcomes. Future research efforts should focus on incorporating diverse perspectives and employing longitudinal and quantitative methods to provide a more comprehensive understanding of prescribing trends and their implications for patient care.

Physician behaviors illustrated either by the policies that they follow or their discourse lie on the foundations of evidence-based medicine. Interventions for which sufficient and compelling evidence have been gathered make it into policy and into practice. While it is sometimes argued that this evidence is biased – clinical trial selection (37), inadequate study population selection (38), among other – the simpler argument claiming that economically nonprofitable evidence will not be so thoroughly researched and therefore is less likely to become policy or practice remains largely unheard (39). Although nutrition and exercise were indeed mentioned in policy and practice, they were rarely considered as the foundations of bone health. Pharmaceutical treatment is perceived oftentimes as the primary and obvious course of action. The possibility that large amount of resources is spent for the development of evidence supporting pharmaceuticals that then enter the realm of policy and then practice create the possibility for a double suboptimal cost: the development of potentially futile research, which transform into futile or suboptimal practices. This study contributes to building the argument in favor of fairer distribution of research for healthcare and prevention research, focusing on clinical outcomes rather than entrepreneurial interests.

One limitations of this study are the focus on prescription data in the absence of consumption data. This prevents a definitive link between policies and actual prescription patterns. While prescription data provides valuable insights into trends, it does not account for the number of patients ultimately using the prescribed medications or the overall treatment adherence. Without data on consumption rates or the estimated number of users, it is challenging to accurately assess the true impact of policies on treatment uptake. Additionally, the lack of a population denominator limits our ability to contextualize the prescribing trends within the broader population, making it difficult to fully understand the extent to which these trends reflect actual patient care. Future research incorporating consumption data and more precise population metrics would provide a clearer picture of the relationship between policy changes and real-world prescribing behaviors.

While our study provides valuable insights into osteoporosis drug prescribing trends, it has limitations that warrant consideration. The qualitative nature of our analysis limits the generalizability of our findings beyond the contexts of England and France as well as its timeframe. Additionally, the focus on physician perspectives may overlook important insights from patients and other stakeholders. Future research should incorporate diverse perspectives and employ longitudinal and quantitative methods to provide a more comprehensive understanding of prescribing trends. Despite these limitations, our study contributes to the growing body of literature on osteoporosis management and prescribing behaviors. Future research efforts should build on these findings to develop evidence-based strategies for optimizing healthcare resource allocation and improving patient care.

## Data Availability

The raw data supporting the conclusions of this article will be made available by the authors, without undue reservation.

## References

[1] Grob G. *Aging Bones: A Short History of Osteoporosis.* Baltimore, MA: John Hopkins University Press (2014).

[2] Wright N Looker A Saag K Curtis J Delzell E Randall S The recent prevalence of osteoporosis and low bone mass in the United States based on bone mineral density at the femoral neck or lumbar spine. *J Bone Miner Res.* (2014) 29:2520–6. 24771492 10.1002/jbmr.2269 PMC4757905

[3] Li G Thabane L Papaioannou A Ioannidis G Levine M Adachi J. An overview of osteoporosis and frailty in the elderly. *BMC Musculoskelet Disord.* (2017) 18:46. 10.1186/s12891-017-1403-x 28125982 PMC5270357

[4] Conrad P. *The Medicalization of Society : On the Transformation of Human Conditions into Treatable Disorders.* Baltimore, MD: Johns Hopkins University Press (2007).

[5] Mulley G. Stop the medicalisation of old age. *BMJ* (2012) 2012:344.

[6] Abraham J. The pharmaceutical industry, the state and the NHS. In: Gabe J Calnan M eds *The New Sociology of the Health Service.* London: Routledge (2009).

[7] Giangregorio L Papaioannou A Cranney A Zytaruk N Adachi J. Fragility fractures and the osteoporosis care gap: An international phenomenon. *Semin Arthritis Rheum.* (2006) 35:293–305. 10.1016/j.semarthrit.2005.11.001 16616152

[8] Glasziou P Chalmers I. Research waste is still a scandal—an essay by Paul Glasziou and Iain Chalmers. *BMJ.* (2018) 363:k4645. 10.1136/bmj.k4645

[9] Ioannidis JPA. Why most published research findings are false. *PLoS Med.* (2005) 2(8):e124. 10.1371/journal.pmed.0020124 16060722 PMC1182327

[10] Lee R Reher D. Introduction: The landscape of demographic transition and its aftermath. *Popul Dev Rev.* (2011) 37:1–7. 10.3389/fpubh.2023.1126900 21714197

[11] Dall TM Gallo PD Chakrabarti R West T Semilla AP Storm MV. An aging population and growing disease burden will require alarge and specialized health care workforce by 2025. *Health Affairs.* (2013) 32(11):2013–20. 10.1377/hlthaff.2013.0714 24191094

[12] Morgan S. Drug spending in Canada: Recent trends and causes. *Med Care.* (2004) 42(7):635–42. 10.1097/01.mlr.0000129494.36245.4b 15213487

[13] Souccar T. *Le Mythe de l’Ostéoporose*. Thierry Souccar Editions. Vergéze. (2013).

[14] Goldacre B. *Bad Pharma. Fourth Estate.* London. (2012).

[15] Williams S Martin P Gabe J. The pharmaceuticalisation of society? A framework for analysis. *Sociol Health Illness.* (2011) 33:710–25. 10.1111/j.1467-9566.2011.01320.x 21371048

[16] Spiegel A. *How a Bone Disease Grew to Fit the Prescription.* Washington, DC: National Public Radio (NPR) (2009).

[17] Body J Bergmann P Boonen S Bousten Y Bruyere O Devogelaer J Non-pharmacological management of osteoporosis: A consensus of the Belgian Bone Club. *Osteoporos Int.* (2011) 22:2769–88. 21360219 10.1007/s00198-011-1545-x PMC3186889

[18] Buse K Mays N Walt G. *Making Health Policy.* Maidenhead: Open University Press (2005).

[19] Masic I Miokovic M Muhamedagic B. Evidence based medicine - new approaches and challenges. *Acta Inform Med.* (2008) 16:219–25. 24109156 10.5455/aim.2008.16.219-225 PMC3789163

[20] Eisenberg J. Physician utilization: The state of research about physicians’ practice patterns. *Med Care.* (2002) 40:1016–35. 12409848 10.1097/01.MLR.0000032181.98320.8D

[21] Hernlund E Svedbom A Ivergard M Compston J Cooper C Stenmark J Osteoporosis in the European Union: Medical management, epidemiology and economic burden: A report prepared in collaboration with the International Osteoporosis Foundation (IOF) and the European Federation of Pharmaceutical Industry Associations (EFPIA). *Arch Osteoporos.* (2013) 8:136. 10.1007/s11657-013-0136-1 24113837 PMC3880487

[22] OpenPrescribing.net. *Explore England’s prescribing data. EBM DataLab, University of Oxford. https://openprescribing.net/ 2018. Prescribing Trends by Section - items and cost per 1000 population in Chapter 6: Endocrine System. 6.6 Drugs Affecting Bone Metabolism. Consulted 06 June 2024.* (2024).

[23] Open Data. *Open Medic : Base complète sur les dépenses de médicaments interrégimes.* (2018). Available online at: https://www.data.gouv.fr/fr/datasets/open-medic-base-complete-sur-les-depenses-de-medicaments-interregimes (accessed June 6, 2024).

[24] Guillemot J. *Explaining Osteoporosis Drug Prescribing Trends: Qualitative Analyses in England and France.* Doctoral Thesis. London: King’s College London (2020).

[25] Huot L Couris C Tainturier V Jaglal S Colin C Schott A. Trends in HRT and anti-osteoporosis medication prescribing in a European population after the WHI study. *Osteoporosis Int.* (2008) 19:1047–54. 10.1007/s00198-008-0587-1 18373055

[26] Nolte E Corbett J. *International Variation in Drug Usage. An Exploratory Analysis of the “Causes” of Variation. Research Reports.* Santa Monica, CA: RAND Corporation. (2014). 104. 10.7249/rhq-04-04-01 PMC5158257 28083348

[27] Khosla S Shane E. A crisis in the treatment of osteoporosis. *J Bone Miner Res.* (2016) 31:1485–7. 27335158 10.1002/jbmr.2888

[28] World Health Organization. *Introduction to Drug Utilization Research.* Geneva: WHO (2003).

[29] Lewis-Beck M Bryman A Liao T. *The Sage Encyclopedia of Social Science Research Methods.* Thousand Oaks, CA: SAGE (2004).

[30] Miles MB Huberman AM Saldaña J. *Qualitative Data Analysis: A Methods Sourcebook.* 3rd ed. Thousand Oaks, CA: SAGE Publications (2014).

[31] Asch S Connor S Hamilton E Fox S. Problems in recruiting community-based physicians for health services research. *J Gen Intern Med.* (2000) 15:591–9. 10940152 10.1046/j.1525-1497.2000.02329.x PMC1495576

[32] Lavrakas P. *Purposive Sample. Encyclopedia of Survey Research Methods.* Thousand Oaks, CA: Sage Publications (2008).

[33] Liu L. Using generic inductive approach in qualitative educational research: A case study analysis. *J Educ Learn.* (2016) 5:129–35.

[34] Sheffield.ac.uk. *Welcome to FRAX§* . (2024). Available online at: https://www.sheffield.ac.uk/FRAX/ (accessed June 5, 2024).

[35] Narayan S Nishtala P. Population-based study examining the utilization of preventive medicines by older people in the last year of life. *Geriatr Gerontol Int.* (2018) 18:892–8. 29392866 10.1111/ggi.13273

[36] Wysowski D Greene P. Trends in osteoporosis treatment with oral and intravenous bisphosphonates in the United States, 2002-2012. *Bone.* (2013) 57:423–8. 10.1016/j.bone.2013.09.008 24063946

[37] Mayo-Wilson E Li T Fusco N Bertizzolo L Canner J Cowley T. Cherry-picking by trialists and meta-analysts can drive conclusions about intervention efficacy. *J Clin Epidemiol.* (2017) 91:95–110. 10.1016/j.jclinepi.2017.07.014 28842290

[38] Giovanni T Kitty JJ Friedo WD Carmine Z. Selection bias and information bias in clinical research. *Nephron Clin Pract. 1* (2010) 115(2):c94–9. 10.1159/000312871 20407272

[39] Krimsky S. *Science in the Private Interest: Has the Lure of Profits Corrupted Biomedical Research?.* Lanham, MA: Rowman & Littlefield (2004).

